# Care2Caregiver: A Peer Support Helpline for Caregivers of Persons Living With Dementia

**DOI:** 10.1093/geroni/igaa057.1541

**Published:** 2020-12-16

**Authors:** Michelle Zechner, Margaret Swarbrick, Mary-Catherine Lundquist

**Affiliations:** 1 Rutgers, RBHS, Piscataway, New Jersey, United States; 2 Rutgers, PIscataway, New Jersey, United States

## Abstract

Caregivers of persons with Alzheimer’s disease and Related Dementia (CADRD) provide a significant amount of support to their family members, however, experience many challenges and stress that impacts their quality of life, emotional and physical well-being. They also have difficulty accessing services. Peers supporters, or CADRD with specialized training, are an important resource to help alleviate stress related to caregiving and focus on wellbeing, yet few studies have examined peer support. Our retrospective cohort study examined four years of data collected from a peer support helpline for CADRD in a northeastern state. Data included: demographics, presenting issues, services provided to assess for changes across time and call frequency. The Peer Specialists were CADRD who received specialized training and supervision to provide telephonic peer support. Caregivers were primarily female, and the average age of caller increased significantly over time. Issues related to personal emotional wellbeing was the primary reason for the call. The primary type of support provided was peer support interactions, follow-up calls, and links to resources focused on caregiver well-being. Callers reported distress from offering care to multiple recipients. Electronic follow-up (e.g. email and chat) increased over time. Peer support helpline programs have the potential to address the practical, physical, social and emotional needs of CADRD in the location of the caregiver choice. This model has potential to reach the growing number CADRDs via an array of web-based services including chat, email and online support, which can help overcome transportation and other logistical barriers to support.

